# Feasibility of video‐based skill assessment for percutaneous nephrostomy training in Senegal

**DOI:** 10.1049/htl2.12107

**Published:** 2024-12-14

**Authors:** Rebecca Hisey, Fatou Bintou Ndiaye, Kyle Sunderland, Idrissa Seck, Moustapha Mbaye, Mohammed Keita, Mamadou Diahame, Ron Kikinis, Babacar Diao, Gabor Fichtinger, Mamadou Camara

**Affiliations:** ^1^ Laboratory for Percutaneous Surgery Queen's University Ontario Canada; ^2^ Ecole Supérieure Polytechnique Dakar Senegal; ^3^ Surgical Planning Laboratory Harvard Medical School Boston Massachusetts USA; ^4^ Université Cheikh Anta Diop Dakar Senegal; ^5^ Present address: 21‐25 Union St. Kingston ON Canada K7L 2N8

**Keywords:** medical computing, motion measurement, object detection, tracking

## Abstract

Percutaneous nephrostomy can be an effective means of preventing irreparable renal damage from obstructive renal disease thereby providing patients with more time to access treatment to remove the source of the blockage. In sub‐Saharan Africa, where there is limited access to treatments such as dialysis and transplantation, a nephrostomy can be life‐saving. Training this procedure in simulation can allow trainees to develop their technical skills without risking patient safety, but still requires an ex‐pert observer to provide performative feedback. In this study, the feasibility of using video as an accessible method to assess skill in simulated percutaneous nephrostomy is evaluated. Six novice urology residents and six expert urologists from Ouakam Military Hospital in Dakar, Senegal performed 4 nephrostomies each using the setup. Motion‐based metrics were computed for each trial from the predicted bounding boxes of a trained object detection network, and these metrics were compared between novices and experts. The authors were able to measure significant differences in both ultrasound and needle handling between novice and expert participants. Additionally, performance changes could be measured within each group over multiple trials. Conclusions: Video‐based skill assessment is a feasible and accessible option for providing trainees with quantitative performance feedback in sub‐Saharan Africa.

## Graphical abstract

With limited access to dialysis and transplantation in sub‐Saharan Africa, training for life‐saving procedures like percutaneous nephrostomy is essential to prevent renal damage from obstructive disease. In this study, we used a trained object detection network to compute motion‐based metrics in order to assess differences in ultrasound and needle handling between novices and experts. We demonstrate that video‐based skill assessment can provide accessible, quantitative feedback for nephrostomy training in resource‐limited settings.

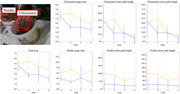

**Abbreviations**
OSATS: Objective Structured Assessment of Technical Skills
RMIS: Robotic minimally invasive surgery
ABS: Acrylonitrile butadiene styrene
YOLO: You‐only‐look‐once
mAP: Mean average precision
IoU: Intersection over union.

## 1 INTRODUCTION

Percutaneous nephrostomy is a critical procedure in the management of various urological conditions, particularly in situations where normal urine flow is obstructed. This procedure involves the insertion of a catheter through the skin into the renal pelvis to drain urine directly from the kidneys. While its primary indication is obstructive renal disease, which can lead to kidney function impairment if untreated, nephrostomy is also utilized in other clinical scenarios, such as the management of certain kidney stones, infections, and injuries requiring temporary diversion of urine.

In sub‐Saharan Africa, the impact of obstructive renal disease is particularly severe due to the higher prevalence of tropical diseases such as schistosomiasis, coupled with rising rates of hypertension and diabetes–both of which can compromise kidney function [1, 2]. Acute obstructive renal failure, most common among individuals aged 60 to 88, represents a significant portion of hospital admissions in this region [3]. Timely intervention with procedures like nephrostomy is crucial, especially given the limited access to advanced treatments like dialysis and kidney transplants. In countries such as Senegal, where dialysis machines are scarce and concentrated in urban areas, nephrostomy often serves as a life‐saving measure, providing patients with the necessary time to seek further treatment. Although Senegal witnessed its first kidney transplant in December 2023, this milestone highlights the ongoing challenges in accessing comprehensive renal care [4].

Though percutaneous nephrostomy is more accessible than most surgical options, novice physicians face a steep learning curve and high complication rates, compromising patient safety [5]. In Senegal, medical training encounters numerous barriers, including limited financial and material resources, and a lack of advanced medical infrastructure. Budgetary constraints lead to less interactive training, resulting in skill gaps for safely performing percutaneous nephrostomy [6]. While simulation‐based training is common in many countries to minimize patient risk, implementing it in Senegal faces challenges, particularly in cost and a shortage of trained experts for providing feedback. Feedback is crucial for trainees' improvement, but with very few trained urologists in Senegal, diverting them from patient care to observe simulations further restricts patient access to care. These obstacles highlight the necessity for automated feedback methods.

Several attempts have been made to automatically assess trainee skill using motion‐based metrics from conventional tracking systems such as optical and electromagnetic (EM) tracking [7, 8, 9]. A notable 2017 study on skill assessment metrics for several ultrasound‐guided needle interventions using EM tracking found that many of the common motion‐based metrics that could be computed from tracking were redundant and loaded onto only two latent factors [10]. Holden et al. deemed these latent factors “probe hand efficiency” and “needle hand efficiency.” While conventional tracking may be effective, Holden et al.'s study demonstrated that we do not necessarily need precise tracking information; we only need metrics that are representative of the efficiency in movement for both the ultrasound probe and needle. We hypothesize that using object detection, we can compute metrics that can measure these two factors from two‐dimensional data only. Object detection from video can provide two‐dimensional tracking‐like information by predicting object location in sequential frames. It can be done using affordable consumer‐grade cameras and does not require any modifications be made to the surgical tools. Previous studies on central venous catheterization have demonstrated that object detection methods can provide skill assessments comparable to those obtained with EM tracking [11]. Furthermore, it has also been recently shown that two‐dimensional projections of motion can be as effective as full three‐dimensional motion for the purposes of skill assessment [12].

Recent advancements in video‐based surgical skill assessment have demonstrated the potential of various machine learning techniques to evaluate procedural competence. Hira et al. [13] employed temporal convolutional networks to assess capsulorhexis skills from video data, offering a streamlined approach to classify surgical performance. Funke et al. [14] utilized 3D convolutional neural networks (ConvNets) to predict skill scores on a modified Objective Structured Assessment of Technical Skills (OSATS) framework, particularly within the context of the JIGSAWS dataset. Similarly, Wang et al. [15] developed a pipeline that combines video‐based methods with surgical gesture recognition to predict skill scores and provide feedback, while Kitaguchi et al. [16] used 3D ConvNets for spatiotemporal analysis to classify surgical skill into different proficiency levels.

However, despite these advancements, the field still faces significant limitations. Many of these studies rely heavily on data from laparoscopic or robotic minimally invasive surgery (RMIS) procedures, which benefit from the availability of extensive training datasets. This abundance of data is not as prevalent in open or percutaneous procedures, such as nephrostomy, where scene complexity and variability present additional challenges. Furthermore, the current methods often focus on binary or categorical classifications of skill, lacking the granularity required to track learning curves or provide detailed, actionable feedback to trainees. These limitations highlight the need for more adaptable and comprehensive approaches that can be applied across a broader range of surgical procedures and training scenarios.

In this article, we introduce and validate a novel approach for assessing skill using video in simulated nephrostomy training in Senegal. Our method aims to the explainability of traditional 3D tracking metrics with the accessibility offered by deep learning‐based video analysis. By leveraging two‐dimensional video data captured by consumer‐grade cameras, we monitor the position and movement of the needle during the procedure, employing object detection algorithms to generate interpretable metrics that reflect technical performance. Additionally, we describe a preliminary version of a low‐cost, open‐source system for ultrasound‐guided percutaneous nephrostomy training, known as the Nephrostomy Tutor. While this system currently serves as a data collection platform, it is designed to evolve into a comprehensive training tool as we validate and integrate the methods of assessment presented here.

## 2 METHODS

### 2.1 System design

**FIGURE 1:**
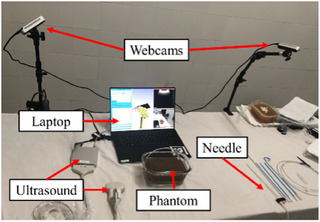
Hardware components of the Nephrostomy tutor system.

The Nephrostomy Tutor system, depicted in Figure 1, is composed of a consumer‐grade laptop, a Telemed C5 curvilinear ultrasound probe, and two Intel Realsense D415 webcams mounted on either side of the laptop. The webcams recorded video at a resolution of 640x480 pixels with a frame rate of 10 frames per second. The needle used was a standard 21‐gauge nephrostomy needle, which is commonly employed in clinical practice for this procedure.

One of the key advantages of the Nephrostomy Tutor system is its cost‐effectiveness and adaptability. Built on free, open‐source software platforms, including 3D Slicer (www.slicer.org) and the Plus toolkit (www.plustoolkit.org), the system can interface with a variety of hardware components, such as different ultrasound devices and webcams. While this study utilized a Telemed ultrasound and Intel Realsense cameras, the abstraction layer provided by the Plus toolkit offers flexibility in hardware selection, allowing the system to be tailored to various budgets and preferences, making it particularly suitable for low‐resource settings like those in sub‐Saharan Africa.

**FIGURE 2:**
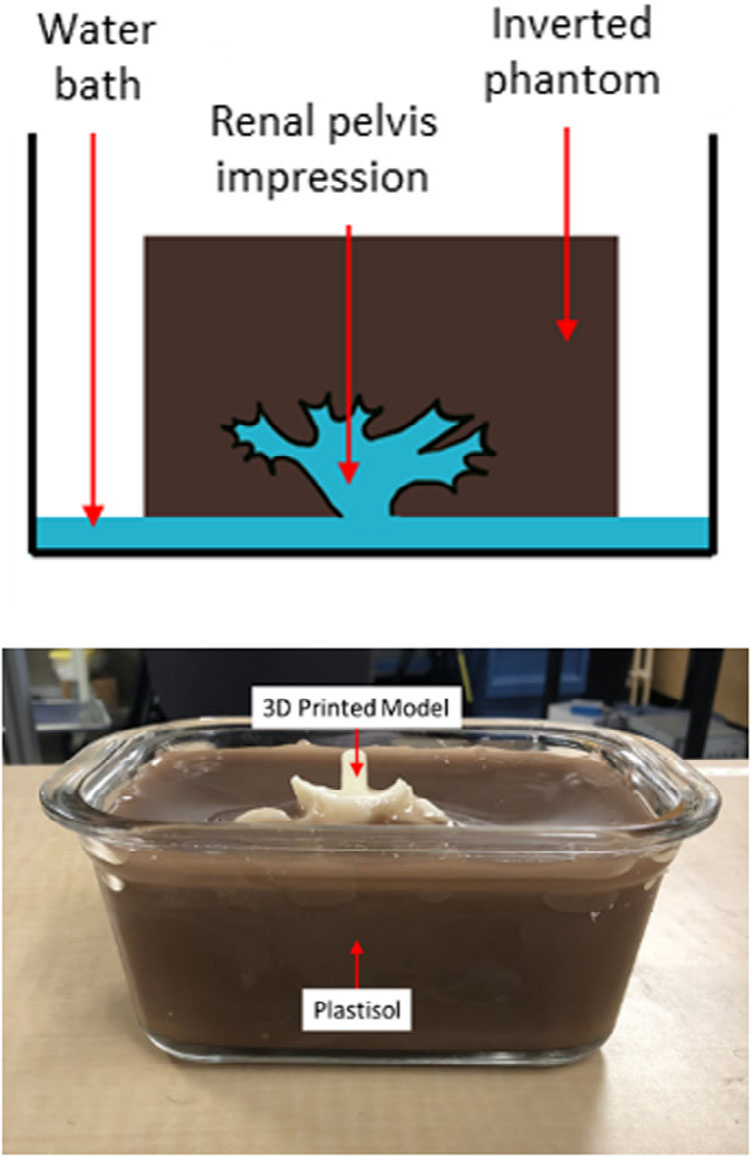
Schematic of renal pelvis phantom (Top), and renal pelvis phantom prior to removing 3D printed calyx model (Bottom).

The final component of the system is a low‐cost kidney cavity phantom on which the participants can practice inserting the nephrostomy into the kidney calyxes. Made of plastisol with added cellulose to mimic tissue properties under ultrasound, the phantom features a cavity created using a mold segmented from a computed tomography scan and 3D printed in Acrylonitrile Butadiene Styrene (ABS) plastic. After the plastisol hardens, the calyx mold is removed, and the phantom is inverted into a water bath and shaken to remove any air bubbles trapped in the calyx cavity. The low‐cost renal pelvis phantom is shown in Figure 2.

### 2.2 Dataset

Six novice urology residents and six expert urologists from Ouakam Military Hospital in Dakar, Senegal performed four trials of percutaneous nephrostomy each using the Nephrostomy tutor setup. The trials recorded the two color video streams from the webcams along with the ultrasound video. The recordings ranged in length from 105 s to 672 s and were recorded at approximately 10 frames per second due to hardware constraints.

To train the object detection network, each color video was divided into individual frames. All frames from the first trial from each participant were manually annotated with the bounding box locations of the ultrasound probe and needle. This amounted to 97,949 images including 78,241 ultrasound probe annotations and 44,685 needle annotations that could be used for training the object detection network.

### 2.3 Object detection network

The object detection network that was selected for this study was the small version You‐Only‐Look‐Once version 8 (YOLOv8s). This model was selected as it is small and lightweight and is capable of producing predictions quickly, even on CPU. Various YOLO models have also been validated previously for their use in computer‐assisted skill assessment for a variety of open and percutaneous procedures such as central venous catheterization and suturing skills [11, 17].

The network was trained using a leave‐two‐user‐out cross validation scheme, whereby all data from one novice participant and one expert participant were reserved for testing. All data for another novice and expert participant were reserved for validation in each fold and all remaining data was used for training. This folding process repeats until each user has been included in the test set once. Early stopping and data augmentations including random rotation, cropping, color distortions and mosaicking were also used to prevent model overfitting. The network was also initialized with pre‐trained weights from the COCO dataset to ensure robust performance and all images were normalized prior to being passed to the model.

To measure the performance of the object detection network, we computed the mean Average Precision (mAP) for both the ultrasound probe and the needle, as well as an average across all classes. mAP represents the average area under the precision‐recall curve for all classes. We used an Intersection over Union (IoU) threshold of 50% to determine which bounding boxes were considered correct. An IoU threshold of 50% strikes a balance between precision (how accurate the predicted bounding box is) and recall (how well the object detector identifies all objects). An IoU of 50% means that the predicted bounding box needs to overlap significantly with the ground truth box, but it allows for some tolerance in the exact positioning and size of the bounding box. We selected mAP50 as the evaluation metric because it is a widely adopted standard in object detection literature dating back to the PASCAL VOC challenge [18].

### 2.4 Skill assessment metrics

Once the object detection network was trained, predicted bounding boxes were produced for all videos, including those that were not annotated. To simplify the calculation of the skill assessment metrics, only the bounding box with the highest confidence for each class was kept for each frame. From the predicted bounding boxes, we computed the following five skill assessment metrics for each video.

**Total procedure time**: The total procedure time is given by the total length of the recording in seconds. The right and left videos were synchronized, so this value is the same across both webcam videos.

**Tool usage time**: We define the tool usage time as the number of frames in which the object detection network predicts the tool to be visible, divided by the frame rate. This metric is measured in seconds and is computed for both the ultrasound probe and the needle.

**Center path length**: The center path length is defined as the sum of the Euclidean distances (measured in pixels) between the center point of a bounding box for a given tool and the center point of the bounding box of the same tool in the next frame in which it is visible. This metric is calculated across the entire duration of the procedure, thereby capturing the total distance traveled by the tool throughout the procedure. The center path length provides a measure of translational movement as well as some rotational movement, reflecting a cumulative estimation of the movement of the tool over time.

**Corner path length**: The corner path length between sequential frames is defined as the sum of the Euclidean distances between the four corner points of the bounding box for a given tool and the corresponding corner points of the bounding box of the same tool in the next frame in which it is visible. The total corner path length is calculated by summing all corner path lengths across the entire video, thus accounting for the total distance traveled by the tool during the entire procedure. This metric reflects the cumulative movement of the tool, including any rotational and translational movements, providing a detailed assessment of the tool's trajectory. Given that each trial included one video from each of the two webcams, the metric value for each trial was given by the maximum value from the two videos.

### 2.5 Experiments

#### 2.5.1 Novices vs. experts

To determine if the skill assessment metrics can measure differences between novices and experts, we compute the metrics for each trial and compare the values of each metric for trials recorded by experts against the values of each metric produced by novice trials using an independent t‐test.

#### 2.5.2 First trial vs. last trial

Comparing the changes in each metric from a participant's first trial to their last allows us to determine if the skill assessment metrics are sensitive enough to measure changes in performance as a participant learns. For this experiment, we divide the data into trials recorded by experts and trials recorded by novices. For each group, we perform a paired t‐test to measure the change in the value of each metric from the first trial to the last.

### 2.6 Study design considerations

This study was designed to evaluate the technical feasibility of video‐based skill assessment that will be integrated into the Nephrostomy Tutor system. As the primary objective was to assess whether this approach can successfully differentiate between novice and expert performance based on motion‐based metrics, no formal training or blinding procedures were implemented. Participants were familiarized with the system's operation and with the procedure, but no specific training sessions were provided, as the study did not aim to evaluate user experience or usability. These aspects will be addressed in future studies, where we plan to gather detailed usability feedback and evaluate the system's effectiveness as a comprehensive training tool.

## 3 RESULTS

### 3.1 Object detection network

YOLOv8s was able to detect the ultrasound probe and the needle with an average mAP50 of 0.70±0.12 across all folds. The ultrasound probe had the highest mAP50 of the two classes at 0.79±0.12, while the needle had lower performance with an average mAP50 of 0.61±0.15 across all six folds.

### 3.2 Skill assessment metrics

**FIGURE 3:**
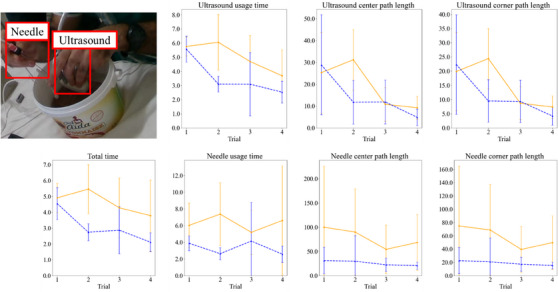
Average learning curves for all skill assessment metrics. The *y*‐axis is normalized by dividing all values from the minimum expert value for each respective metric.

The total procedure time, along with the usage time, center path length and corner path length were computed for each trial from all participants. The metrics computed for the novice trials were higher than those for the expert trials across all metrics. The learning curves for each participant group across the four trials are shown in Figure 3.

### 3.3 Experiments

#### 3.3.1 Novices vs. Experts

**FIGURE 4:**
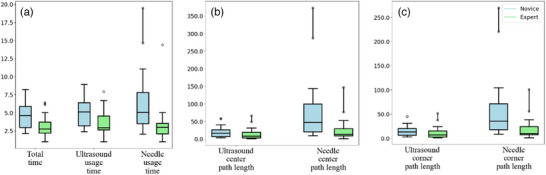
Distribution of values for the novice and expert groups for the path length metrics. (a) represents the distribution of the time‐based metrics, (b) represents the distribution of the center distances and (c) represents the distribution of the corner distances. The *y*‐axis is normalized by dividing all values from the minimum expert value for each metric.

There were statistically significant differences between novices and experts for all time‐based metrics, including: total procedure time and usage time for both the ultra‐sound and needle. Total procedure time had the largest difference between novices and experts (t = 3.20, p = 0.003). The needle usage time had the next largest difference (t = 2.82, p = 0.007), followed closely by the ultrasound usage time (t = 2.69, p = 0.01). The distribution of values for the novice and expert groups for the time‐based metrics are shown in Figure 4a.

Neither of the path length metrics showed a significant difference between novices and experts for the ultrasound probe. The ultrasound corner path length had a marginally larger difference (*t* = 1.03, *p* = 0.31) compared to the ultrasound center path length (*t* = 0.99, *p* = 0.33). Both needle path length metrics showed significant differences between the novice and expert groups. Once again, the needle corner path length had a marginally larger difference (*t* = 2.51, *p* = 0.02) between the two groups when compared to the center path length (*t* = 2.43, *p* = 0.02). The distribution of values for the novice and expert groups for the path length metrics are shown in Figures 4b and 4c.

#### 3.3.2 First trial vs. last

The ultrasound usage time was the only metric in which the novice participants had a significant difference between their first trial and their last (*t* = 2.66, *p* = 0.04). There was no difference in the needle usage time between the first and last trial (*t* = −0.01, *p* = 0.99). The metric values for the last trial were lower for the total procedure time (*t* = 1.25, *p* = 0.27), ultrasound center path length (*t* = 2.00, *p* = 0.10) and the ultrasound corner path length (*t* = 2.04, *p* = 0.09) for the novice group, but these differences were not significant. The needle center path length (*t* = 0.64, *p* = 0.55) and needle corner path length (*t* = 0.70, *p* = 0.52) decreased slightly from the first trial to the last trial for the novice group, but again these differences were not significant.

The expert group showed significant decreases from the first trial to the last in the total procedure time (*t* = 5.99, *p* = 1.8e‐3) and the usage times for both the ultra‐sound probe (*t* = 11.04, *p* = 1.0e‐4) and needle (*t* = 2.64, *p* = 0.05). All path length metrics showed decreases from the first trial to the last for the expert group, though the differences were not significant (*p* > 0.10). The distribution of values in the first and last trial for both novice and expert groups can be seen in the learning curves in Figure 3.

## 4 DISCUSSION

This study represents a significant advancement in the application of video‐based skill assessment for percutaneous nephrostomy, a critical procedure in managing obstructive renal disease, particularly in low‐resource settings. Utilizing the YOLOv8s model, we were able to recognize the ultrasound probe and needle reasonably well, despite some challenges in consistently detecting the needle due to its small size and variable positioning. These challenges are not uncommon in applying 2D object detection to surgical tools, but we anticipate that performance will improve as more annotated data becomes available.

Our findings reveal that experts completed procedures significantly faster than novices, with marked differences in total procedure time, ultrasound usage time, and needle usage time. Total procedure time is a key indicator of procedural efficiency in clinical practice. Shorter procedure times are generally associated with reduced patient discomfort, lower complication rates, and improved overall outcomes. The significant differences observed in tool usage time between experts and novices underscore the importance of dexterity and confidence in using the ultrasound probe and needle. Prolonged usage times among novices suggest a lack of familiarity or hesitation, which can lead to increased patient risk and procedural complications.

Although no significant differences were observed in ultrasound probe path length metrics, novices demonstrated a steep learning curve, rapidly approaching expert levels in ultrasound skills over four trials. In contrast, novices consistently exhibited higher values in needle path length metrics across all trials, indicating that mastering needle placement into the calyx requires more time and practice. The path length metrics, particularly for the needle, provide insight into the precision and control of tool movements. Shorter path lengths are indicative of smoother, more deliberate movements, which are essential for minimizing tissue damage and accurately targeting the calyx.

The skill assessment methods employed in this study offer a novel approach by balancing the explainability of traditional 3D tracking metrics with the accessibility provided by deep learning‐based video analysis. This approach is particularly significant given that the entire study was conducted in a low‐resource setting with Senegalese urologists and residents as participants. The ability to compute skill assessment metrics using only consumer‐grade cameras and open‐source software highlights the system's potential for broader adoption in similar low‐resource environments.

While this study used the maximum value for each metric from the two cameras, and metrics were generally higher from the camera on the right‐hand side, further testing is required to determine the impact of factors such as participant handedness. Additionally, the small number of participants and trials represents a limitation of the study. However, given that the current participants included all available urologists and residents at Ouakam Military Hospital, this study provides a valuable foundation for future research. Expanding this work to additional centers and participant cohorts will be crucial for robust validation of the skill assessment metrics and a deeper understanding of longitudinal learning curves.

The current implementation of the Nephrostomy Tutor system involves post‐trial processing of skill assessment metrics, a necessary step for creating and validating a specialized dataset for percutaneous nephrostomy procedures in sub‐Saharan Africa. Moving forward, the integration of real‐time computation capabilities, leveraging the lightweight YOLO neural network, will enable the system to provide immediate, automated feedback during training sessions. This advancement is expected to significantly enhance the system's utility in training environments by offering trainees instant, actionable feedback that is critical for effective skill development.

Additionally, the application of this system to percutaneous nephrostomy is noteworthy for its focus on a procedure that is vital in regions with limited access to advanced renal care. By utilizing a synthetic phantom made from plastisol mixed with cellulose, we were able to simulate percutaneous nephrostomy with a high degree of realism. The ability to repeatedly melt and reform the phantom allows for the creation of multiple, potentially patient‐specific models, thereby increasing the relevance of this training system to clinical practice. However, it is important to acknowledge the limitations of using a single, static synthetic phantom. Future research should explore the incorporation of a variety of phantom models, including patient‐specific variations, to better simulate the diversity of clinical scenarios encountered in real‐world practice.

Finally, future iterations of the Nephrostomy Tutor system may benefit from the integration of advanced imaging techniques, such as real‐time 3D anatomical visualization facilitated by live ultrasound segmentation using deep learning models as proposed in [19]. Incorporating these methods could enhance needle guidance and provide trainees with a more comprehensive understanding of anatomical structures, further bridging the gap between simulation‐based training and clinical practice. Additionally, to further substantiate the validity of the video‐based assessment approach, future studies should aim to directly compare the results obtained with this system to those generated using traditional EM tracking. This comparison will help clarify the strengths and limitations of our approach relative to established methods, potentially guiding further refinements to the system.

This work demonstrates that it is feasible to assess technical skill using video in a low‐resource setting. By incorporating automatic computation of metrics and leveraging objective, motion‐based analysis, the Nephrostomy Tutor system aligns well with competency‐based frameworks in modern medical training. This approach not only allows trainees to refine their skills in a controlled, risk‐free environment but also facilitates real‐time feedback that correlates with established medical training paradigms. Future studies will focus on further validating this system through expert grading and user evaluations, with the goal of enhancing its impact on physician training and patient outcomes.

## 5 CONCLUSIONS

Video‐based feedback presents an affordable solution for providing feedback to trainees learning percutaneous nephrostomy in low‐resource settings. The metrics presented in this work highlighted significant differences in ultrasound probe and needle handling between experts and novices. Additionally, we could measure performance changes within each group over multiple trials. Overall, these results demonstrate that using object detection from video represents a feasible, affordable option for providing performative feedback for percutaneous nephrostomy training in low resource settings.

## AUTHOR CONTRIBUTIONS

**Rebecca Hisey**: Conceptualization; data curation; formal analysis; investigation; methodology; project administration; supervision; writing—original draft; writing—review and editing. **Fatou Bintou Ndiaye**: Data curation; writing—original draft. **Kyle Sunderland**: Software. **Idrissa Seck**: Data curation. **Moustapha Mbaye**: Data curation. **Mohamed Keita**: Data curation. **Mamadou Diahame**: Data curation. **Ron Kikinis**: Funding acquisition; supervision. **Babacar Diao**: Conceptualization; supervision. **Gabor Fichtinger**: Conceptualization; funding acquisition; supervision; writing—review and editing. **Mamadou Camara**: Conceptualization; supervision; writing—review and editing.

## CONFLICT OF INTEREST STATEMENT

The authors declare no conflicts of interest.

## Data Availability

The data that support the findings of this study are available from the corresponding author upon reasonable request.
